# Supplementary material for: Multi-scale spatial modeling of immune cell distributions enables survival prediction in primary central nervous system lymphoma
Source: iScience. 2023 Jul 10;26(8):107331. doi: 10.1016/j.isci.2023.107331 (PMC10393746; doi:10.1016/j.isci.2023.107331)
Supplement: Document S1. Figures S1–S11 [file mmc1.pdf]

iScience, Volume *26*

Supplemental information

Multi-scale spatial modeling of immune cell distributions enables survival prediction in primary central nervous system lymphoma

Margaretha G.M. Roemer, Tim van de Brug, Erik Bosch, Daniella Berry, Nathalie Hijmering, Phylicia Stathi, Karin Weijers, Jeannette Doorduijn, Jacoline Bromberg, Mark van de Wiel, Bauke Ylstra, Daphne de Jong, and Yongsoo Kim

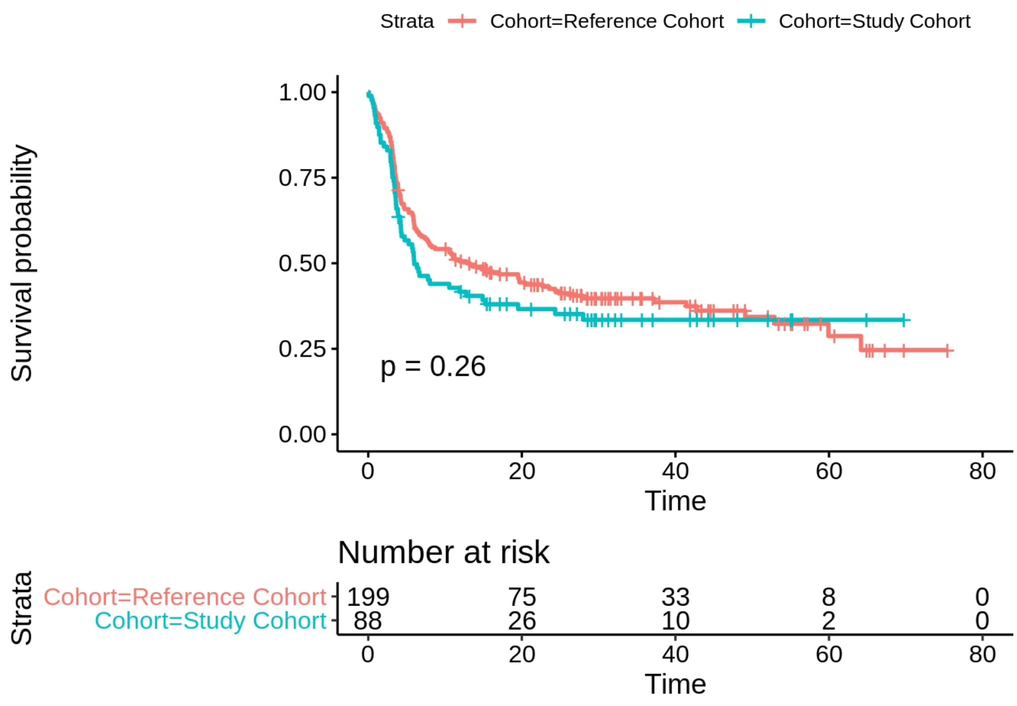

Fig. S1. A comparison of survival outcome of reference (HOVON 105) and study cohort, Related to Table 1. Event-free survival (y-axis) of the entire HOVON 105 cohort (red) and the subset used in this study (blue). Log-rank p-value and number at risk are indicated.

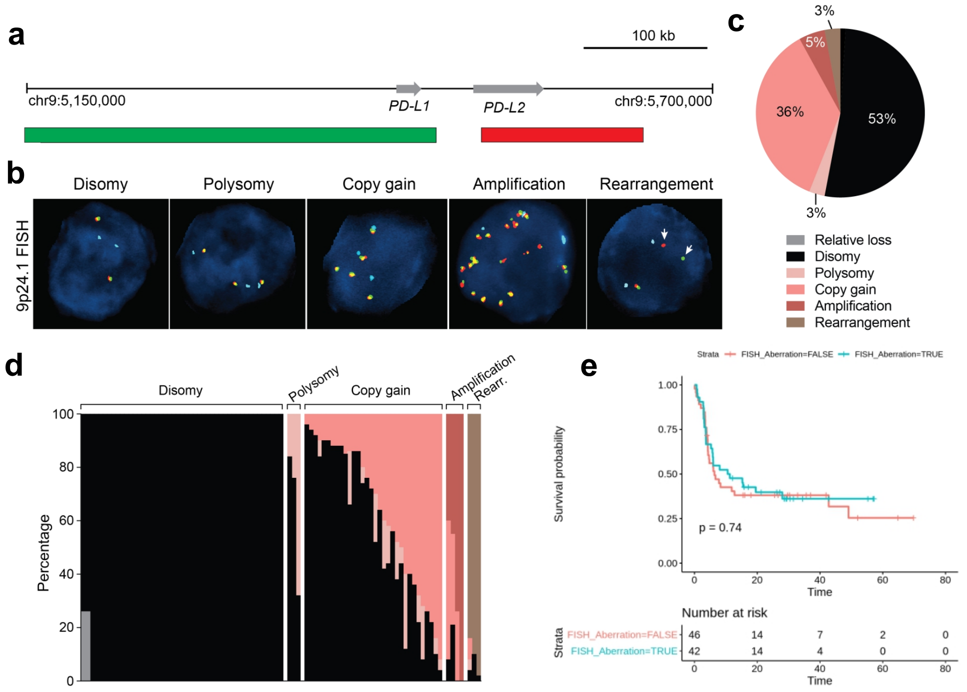

Fig. S2. Fluorescence in situ hybridization (FISH) for the determination of CNAs and translocations of the 9p24.1/*PD-L1/PD-L2* locus, Related to Table 1. a. A graphical representation of the target locus, indicated by green and red bars. **b.** Representative FISH images for each alteration status of 9p24.1/*PD-L1/PD-L2* locus. **c.** A pie chart represents the frequency of the 9p24.1 locus alteration status of the study cohort. **d.** A stacked bar chart represents the frequencies of cells with each 9p24.1/*PD-L1/PD-L2* alteration status in each sample, grouped by sample-wise alteration status of Disomy, Polysomy, Copy Gain, Amplification, and Rearrangement from left to right. **e.** A Kaplan-Meier plot (top) and the table for number at risk (bottom) compare event-free survival of PCNSL samples with (blue) and without (red) 9p24.1/*PD-L1/PD-L2* alteration determined by FISH. Log-rank P-value is indicated at the bottom left of the Kaplan-Meier plot.

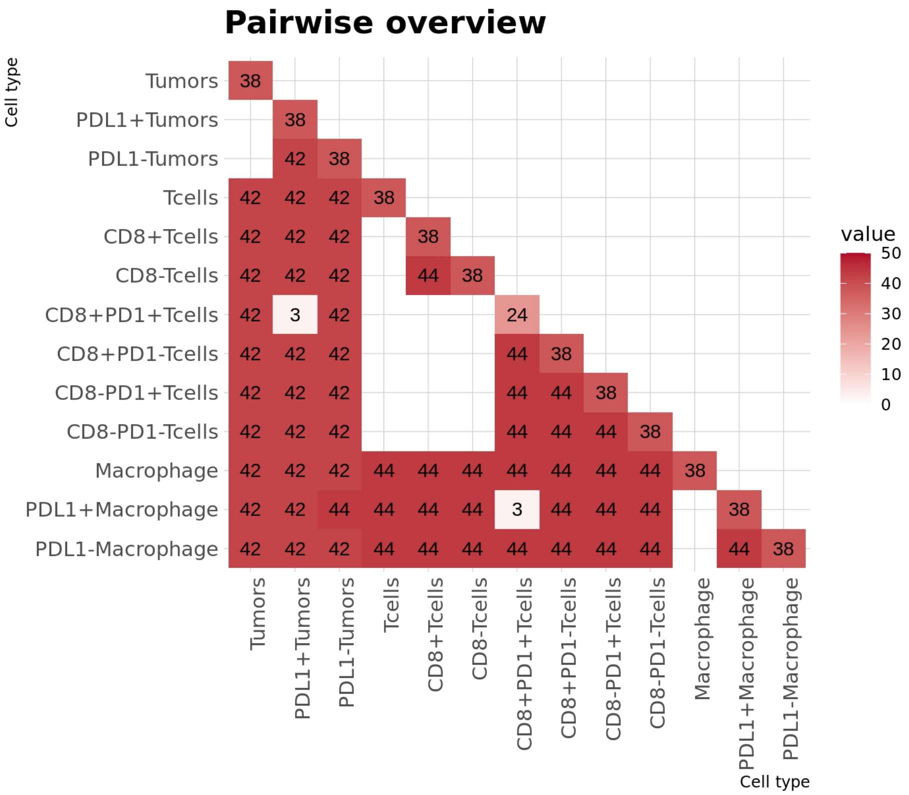

Fig. S3. Number of features per cell type (diagonal) and per pair (lower-triangle), Related to Figure 3. The cell type pairs without the extraction of any features are left empty.

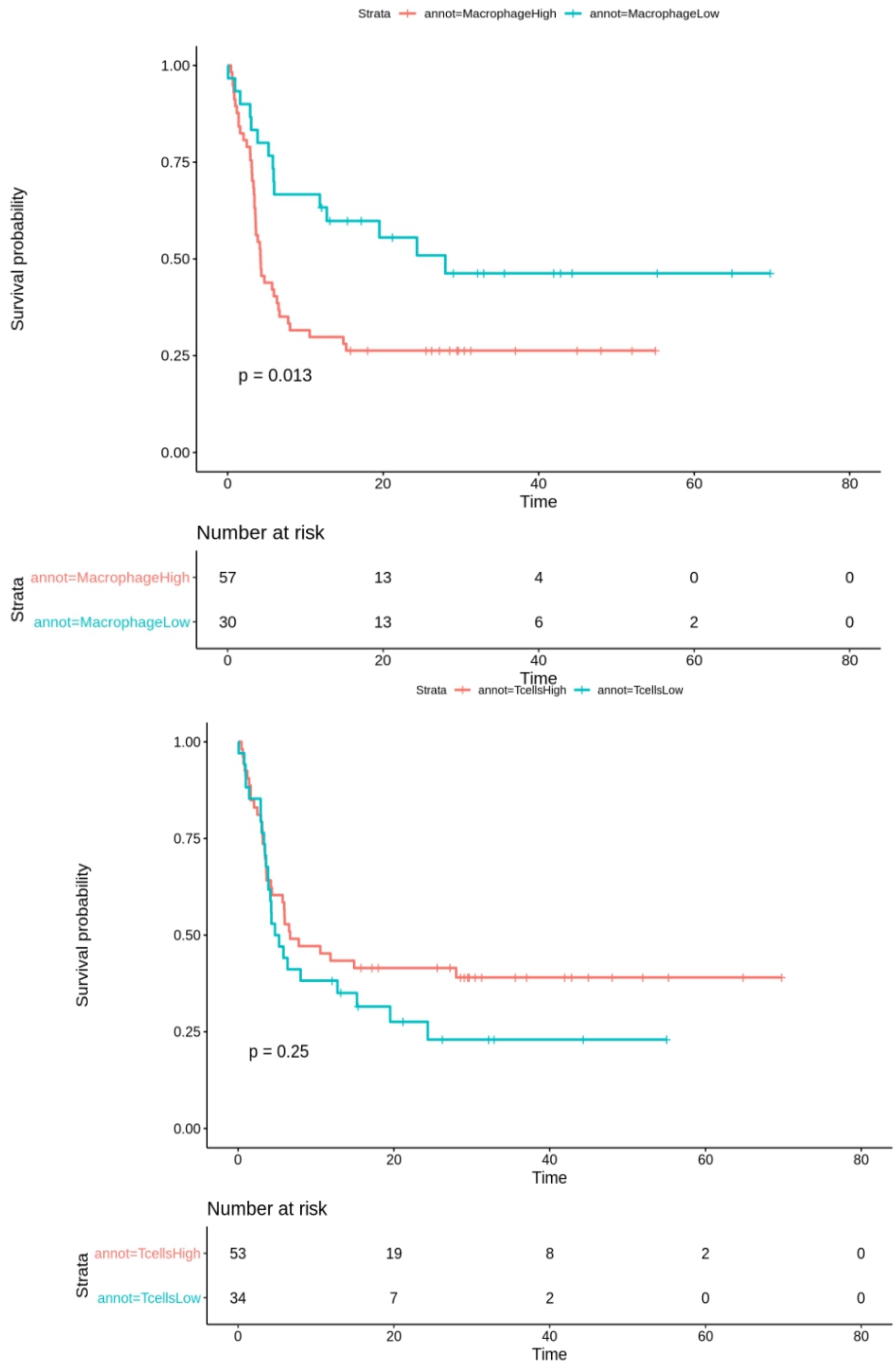

Fig. S4. Survival difference between patients with high/low abundances of macrophages (top) and T cells (bottom), Related to Figure 4. Kaplan-Meier curves to compare the survival of patients classified into macrophage/T cell high/low based on the average cell count. Numbers at risk are shown under the Kaplan-Meier curve. Log-rank P-value is indicated at the bottom left of each Kaplan-Meier curve.

a

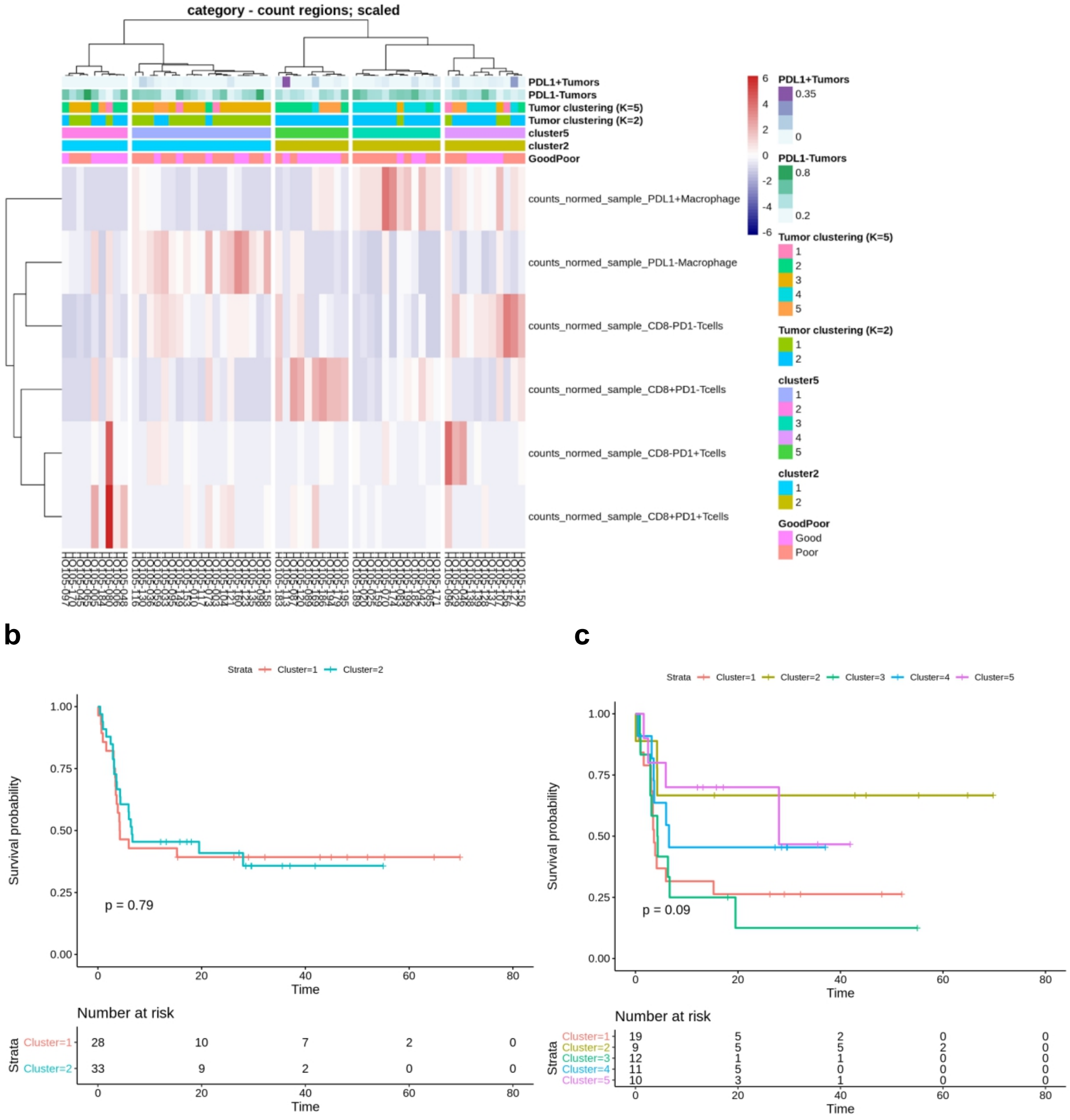

Fig. S5. Unsupervised analysis of non-spatial TME features extracted from the border regions of PCNSL mIF data, Related to Figure 4. a. hierarchical clustering of PCNSL samples using the counts of six non-tumor cell types. The color bars at the top provides annotations for the samples, including the normalized counts of PDL1+ and PDL1- tumors (first two rows), two and five clustering outcomes from tumor images (third and fourth rows) and border images (fifth and sixth rows) and outcome classification with a 12-month threshold (bottom row). **b-c.** Kaplan-Meier plots comparing two (**b**) and five (**c**) clusters generated in (**a**). The numbers at risk are denoted below.

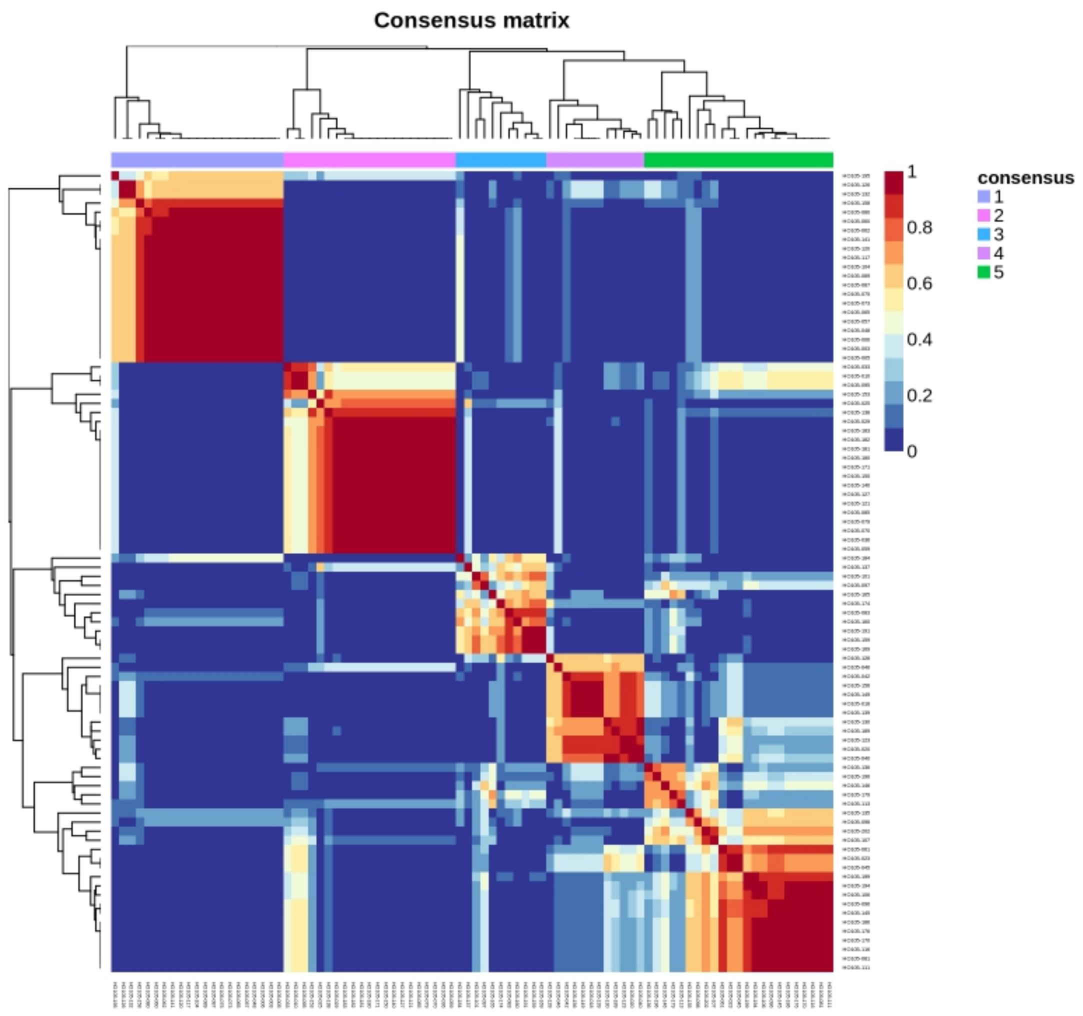

Fig. S6. Consensus clustering analysis of the entire TME features using NMF with K=5, Related to Figure 4. The color denotes the frequency of NMF clustering outcome that clusters each sample pair to the same cluster. Consensus clustering outcome determined by hierarchical clustering is denoted by color bar at the top and dendrograms in rows and columns.

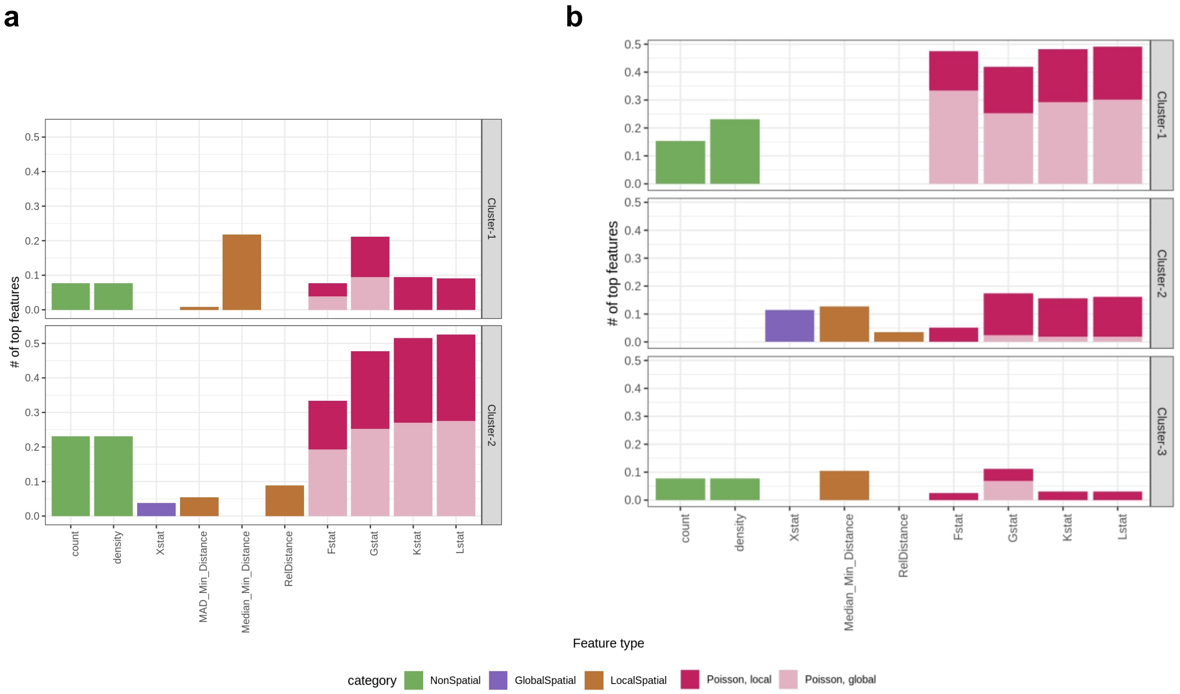

Fig. S7. Frequency of top features of NMF clusters with K=2 and K=3, Related to Figure 4. Normalized frequency of top features (y-axis) for each of the clusters (each panels) derived from the NMF clustering with K=2 (**a**) and K=3 (**b**). The number of top features is normalized by the number of total features for each category and statistics. Bar colors denote the feature types.

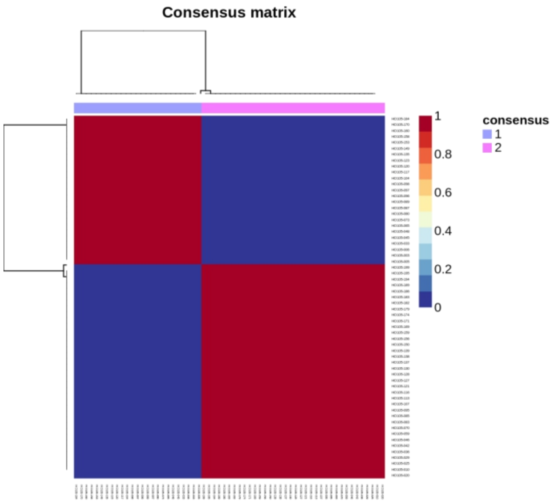
Consensus matrix
consensus
1
2
1
0.8
0.6
0.4
0.2
0

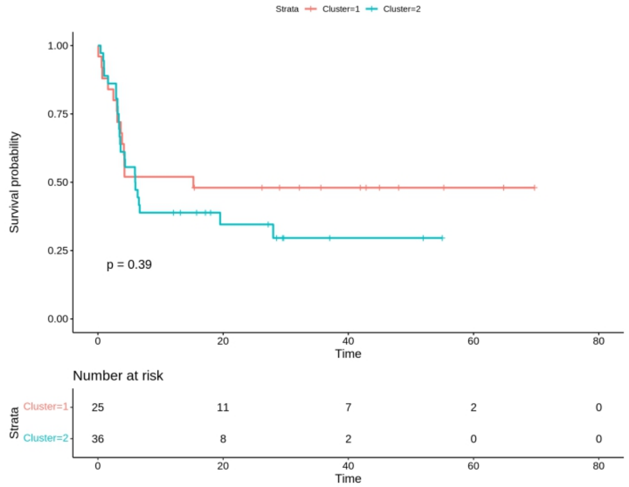
Strata Cluster=1 Cluster=2
Survival probability
1.00
0.75
0.50
0.25
0.00
p = 0.39
0 20 40 60 80
Time
Number at risk
Strata
Cluster=1 25 11 7 2 0
Cluster=2 36 8 2 0 0
0 20 40 60 80
Time

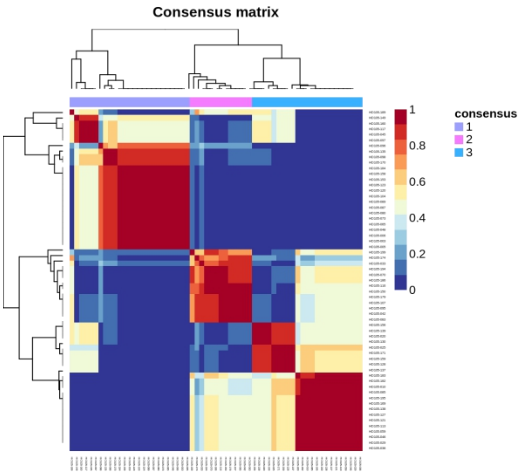
Consensus matrix
consensus
1
2
3
1
0.8
0.6
0.4
0.2
0

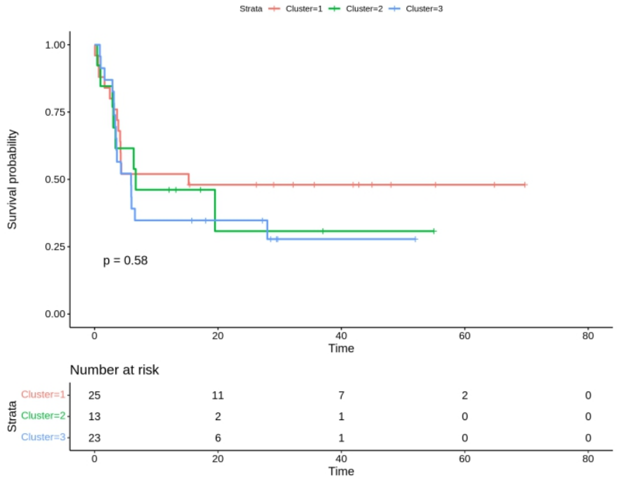
Strata Cluster=1 Cluster=2 Cluster=3
Survival probability
1.00
0.75
0.50
0.25
0.00
p = 0.58
0 20 40 60 80
Time
Number at risk
Strata
Cluster=1 25 11 7 2 0
Cluster=2 13 2 1 0 0
Cluster=3 23 6 1 0 0
0 20 40 60 80
Time

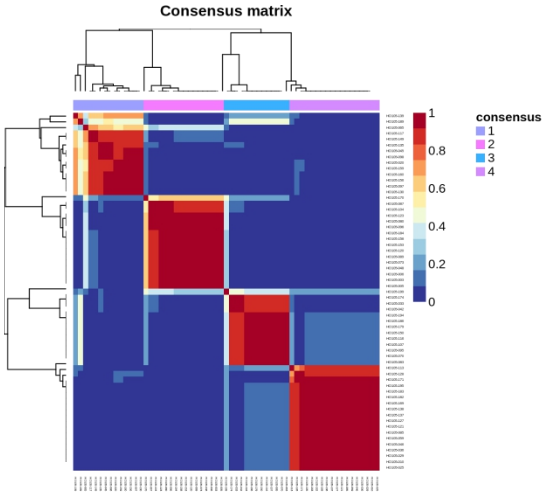
Consensus matrix
consensus
1
2
3
4
1
0.8
0.6
0.4
0.2
0

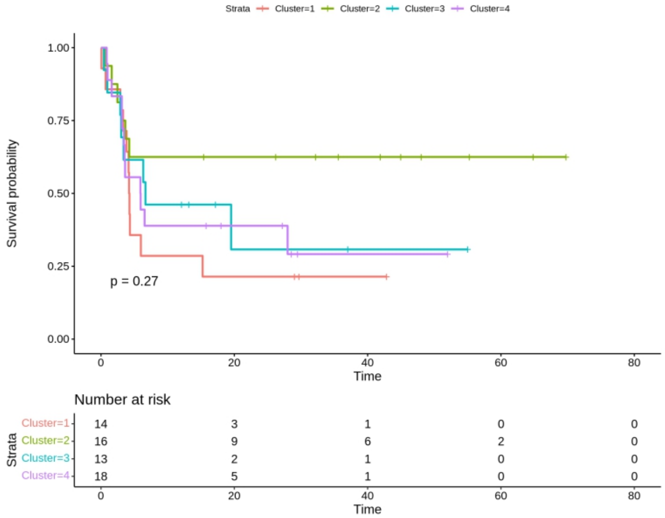
Strata Cluster=1 Cluster=2 Cluster=3 Cluster=4
Survival probability
1.00
0.75
0.50
0.25
0.00
p = 0.27
0 20 40 60 80
Time
Number at risk
Strata
Cluster=1 14 3 1 0 0
Cluster=2 16 9 6 2 0
Cluster=3 13 2 1 0 0
Cluster=4 18 5 1 0 0
0 20 40 60 80
Time

Fig. S8. NMF clustering analysis of spatial and non-spatial TME features extracted from the border regions of PCNSL mIF data, Related to Figure 4. Consensus clustering analysis (left) of the entire TME features using NMF with a different number of clusters: K=2 (top), K=3 (middle), and K=4 (bottom). Kaplan-Meier plots (right) compare the subgroups' survival derived from the NMF clustering. The numbers at risk are denoted under each of the Kaplan-Meier plots.

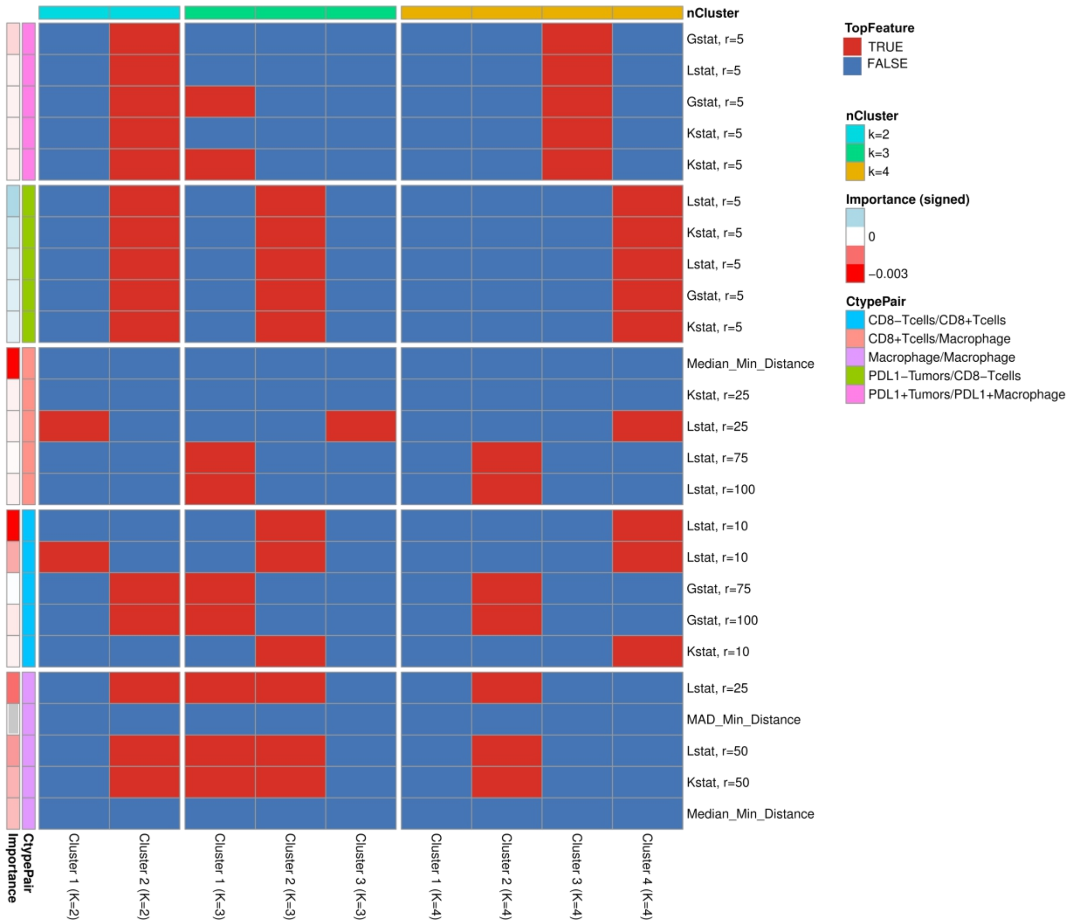

Fig. S9. Involvement of the top spatial features from the RF in the top features for the subgroups identified by unsupervised NMF clustering, Related Figure 5. A heatmap represents the involvement of the top features of the cell types pairs in **Fig. 5e-i** (row) among the top features that define subgroups identified by NMF clustering with K=2 (left), K=3 (middle), and K=4 (right). The signed feature importance and associated cell type pairs are indicated by color bars in the row.

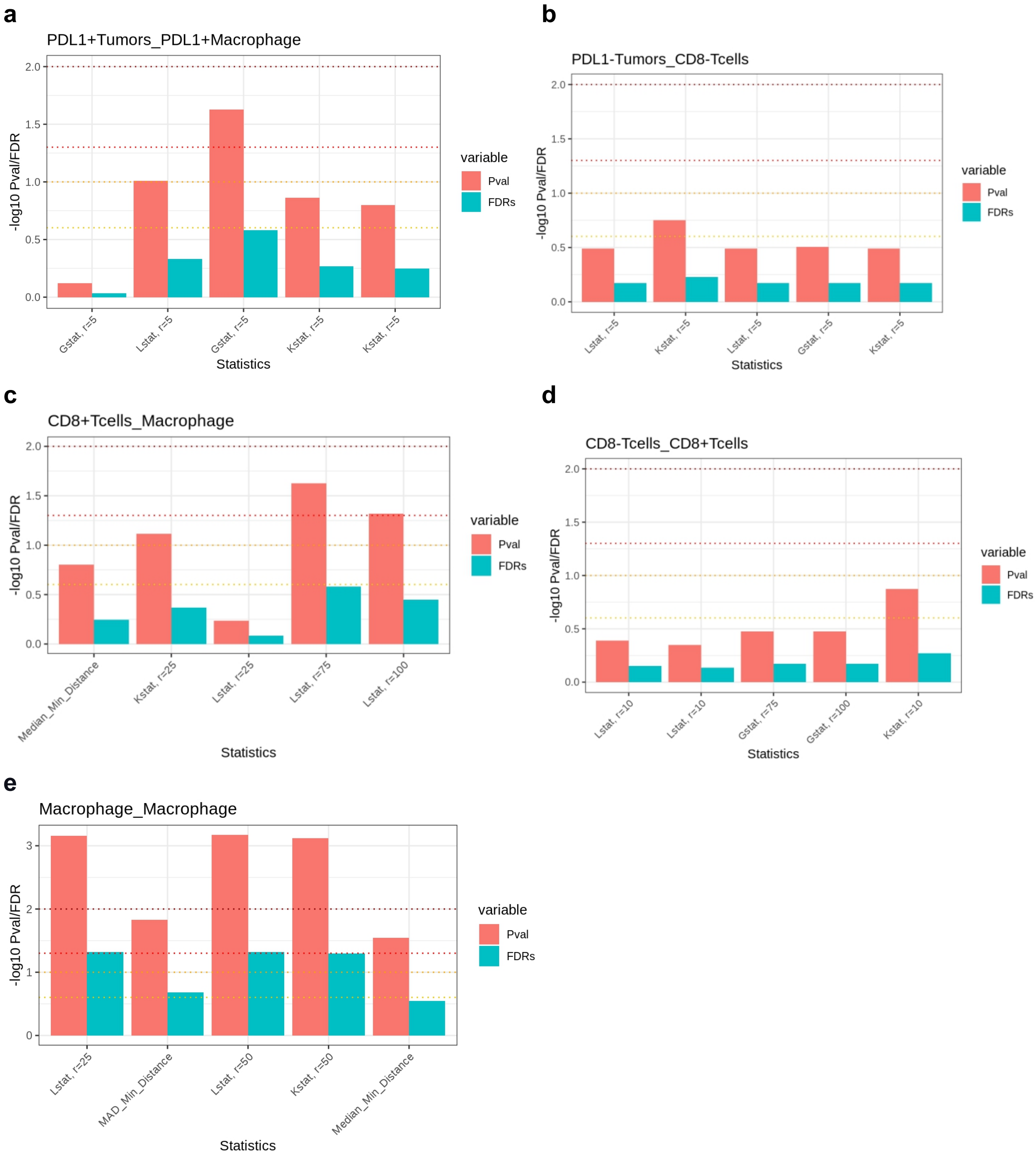

Fig. S10. Univariate Cox regression analysis for the features with the top importance for a subset of cell type pairs, Related to Figure 5. -log10 transformed log-rank p-values (y-axis; red) and False-discovery rates (FDRs; y-axis; blue) from the univariate Cox regression analysis for the features in **Fig. 5e-i**. FDRs were obtained using the p-values from the same Cox regression for the entire 2,980 TME features. Several cutoffs (0.01, 0.05, 0.1, and 0.25) are indicated by the dotted lines (dark red, red, orange and yellow).

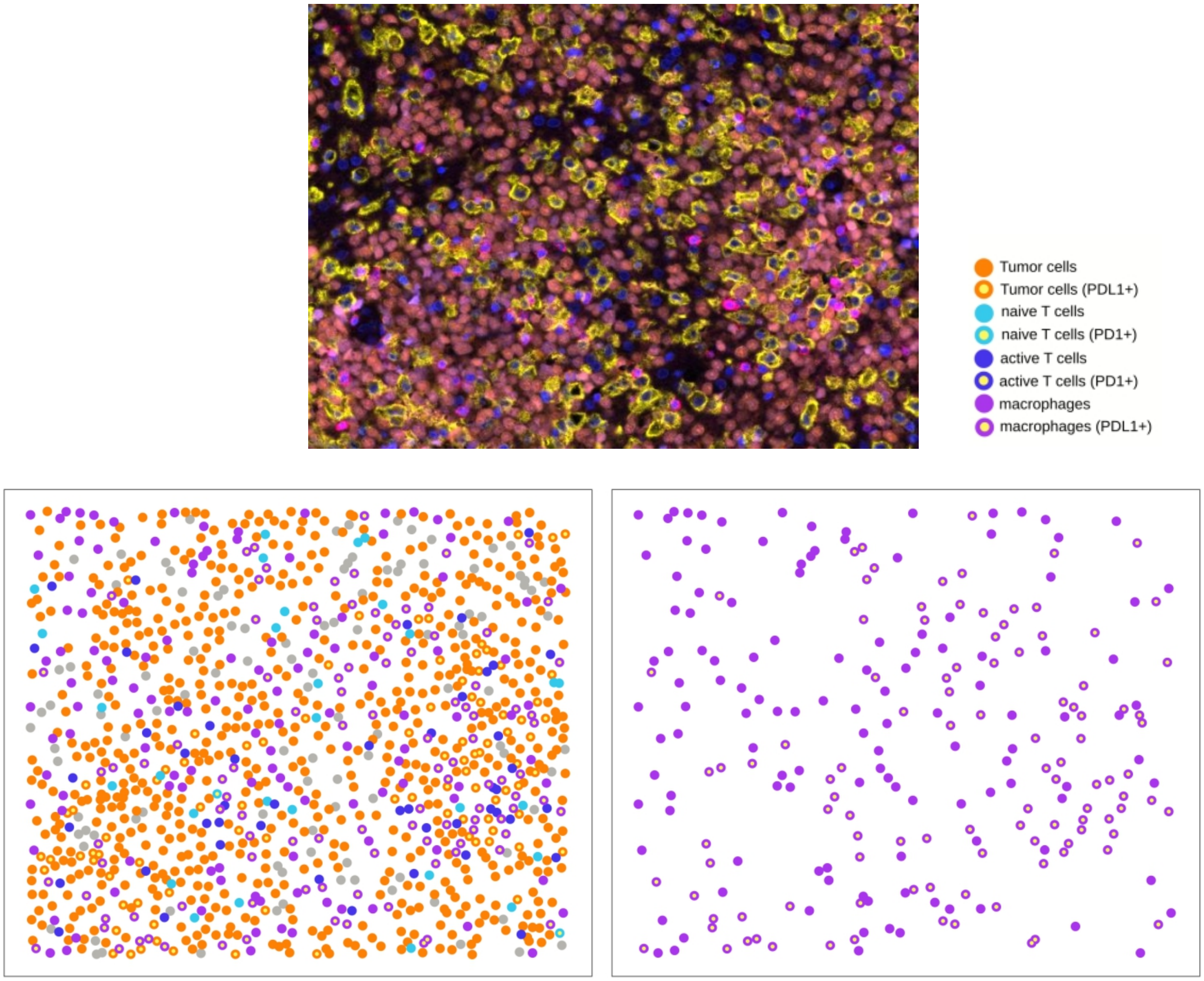

Fig. S11. Example of mIF image with the lowest interaction between macrophage, Related to Figure 6. The raw mIF image (top) and their processed images show all cell types (bottom left) and only macrophage (bottom right). The image has the lowest macrophage interaction according to the L function with the radius of 25.
